# Sarcoidosis: when the initial manifestations are musculoskeletal symptoms

**DOI:** 10.1590/0100-3984.2015.0158

**Published:** 2018

**Authors:** Lara Marinho Reis, Márcio Luís Duarte, Simone Botelho Alvarenga, José Luiz Masson de Almeida Prado, Luiz Carlos Donoso Scoppetta

**Affiliations:** 1 Hospital São Camilo, São Paulo, SP, Brazil; 2 Webimagem, São Paulo, SP, Brazil; 3 Axial Medicina Diagnóstica, Belo Horizonte, MG, Brazil

**Figure 1:**
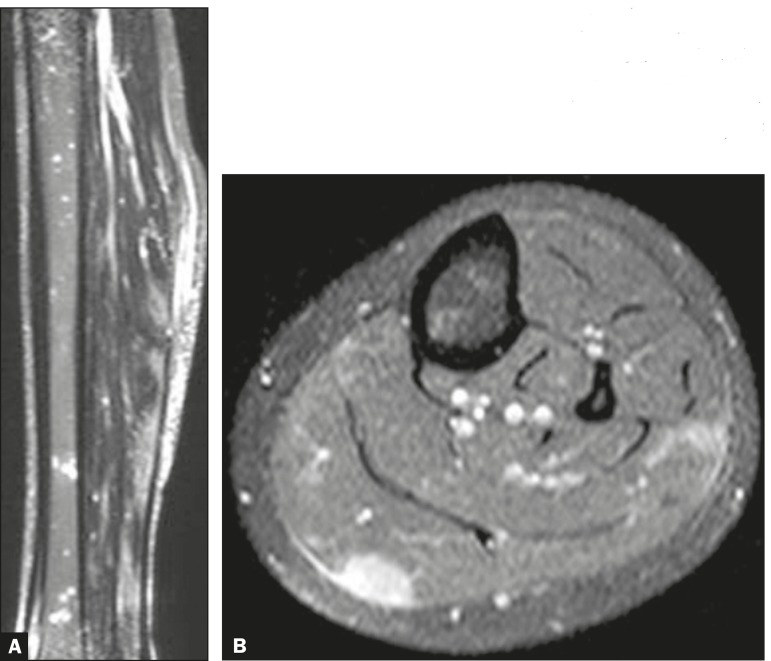
**A:** Non-contrast-enhanced STIR sequence showing nodules of high signal intensity, in the muscle and in the bone. **B:** Contrast-enhanced, fat-saturated T1-weighted sequence showing enhancement of the nodule to be biopsied.

**Figure 2:**
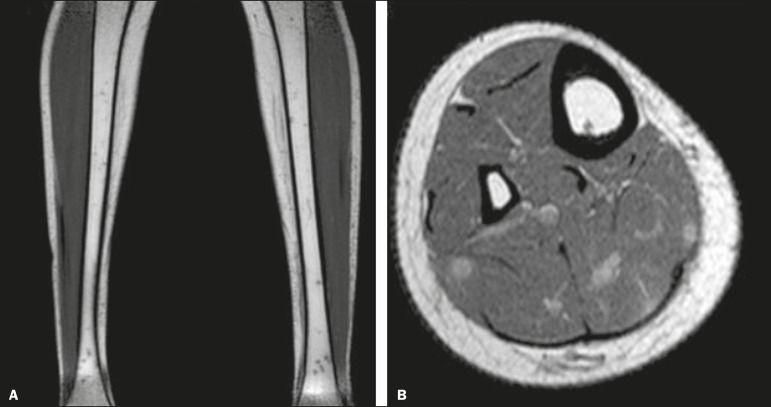
**A:** Non-contrast-enhanced T1-weighted sequence showing nodules of low signal intensity in the bone. **B:** Contrast-enhanced T1-weighted sequence showing enhancement of the nodules in the muscle.

Dear Editor,

A 24-year-old female presented with palpable, painful nodules, which had appeared three weeks prior, on both calves. Laboratory tests showed no abnormalities. Magnetic resonance imaging (MRI) revealed oval lesions showing high signal intensity in short-tau inversion-recovery (STIR) sequences, with enhancement after gadolinium infusion, in the ventral portion of the muscles, together with oval lesions in the bones that showed low signal intensity in T1-weighted sequences and high signal intensity in STIR sequences (Figures 1 and 2). The diagnostic hypothesis was musculoskeletal sarcoidosis, which was confirmed by biopsy. After treatment with corticosteroids, there was regression of the symptoms and of the lesions seen on the MRI scans.

Sarcoidosis is a systemic disease of unknown cause^(1)^, which causes inflammatory granulomas in organs and tissues^(2)^, affecting more women than men^(3)^. Musculoskeletal sarcoidosis is a rare condition, first reported by Licharew in 1908^(4)^, that has two forms of clinical presentation^(2,5)^: nodular and myopathic. The nodular form often involves the extremities, especially the legs^(1)^, giving rise to solitary or multiple nodules^(2)^. The most common sign is a painless or only slightly painful mass^(1,2)^. The myopathic form involves the muscles in a symmetric, diffuse manner and does not form masses, manifesting as slowly progressive myalgia, with weakness, and atrophy^(2)^, as well as resulting in high levels of creatine phosphokinase^(1)^.

Although joint involvement is common in musculoskeletal sarcoidosis, the presence of muscle and bone lesions is not; it is believed that such lesions are indicative of a chronic and prolonged disease course^(6)^. Symptomatic muscle involvement occurs in 1.4% of the known cases of sarcoidosis^(2,7)^, compared with 1.0-13.0% (estimated mean, 5.0%) for symptomatic skeletal involvement^(1)^.

Because of the excellent tissue contrast provided by MRI, it can reveal musculoskeletal changes that are not visible on X-rays, as well as allowing the identification of the lesions that are most suitable for biopsy^(1,8-11)^. On MRI, nodular sarcoidosis has a characteristic appearance that can facilitate its accurate diagnosis-it typically consists of central areas of fibrosis that show low signal intensity in all sequences, together with peripheral areas of granulomas that show high signal intensity in T2weighted sequences and contrast enhancement^(1,6)^-findings collectively known as a "black star". In myopathic sarcoidosis, the MRI findings are nonspecific-the affected muscle shows an increase in signal intensity in T2-weighted sequences^(6)^, also showing atrophy and fatty replacement in some cases^(1)^.

The differential diagnosis of musculoskeletal sarcoidosis includes other benign and malignant mesenchymal masses, such as tophi, pannus formations, and xanthomas^(1)^. In 50-80% of sarcoidosis patients, a biopsy reveals granuloma formation in muscle tissue. However, in most cases, the patient shows no signs or symptoms of the disease^(1)^.
